# Comparing the Performances of Apes *(Gorilla gorilla, Pan troglodytes, Pongo pygmaeus)* and Human Children *(Homo sapiens)* in the Floating Peanut Task

**DOI:** 10.1371/journal.pone.0019555

**Published:** 2011-06-08

**Authors:** Daniel Hanus, Natacha Mendes, Claudio Tennie, Josep Call

**Affiliations:** 1 Department of Developmental and Comparative Psychology, Max Planck Institute for Evolutionary Anthropology, Leipzig, Germany; 2 Department of Social Neuroscience, Max Planck Institute for Human Cognitive and Brain Sciences, Leipzig, Germany; Georgia State University, United States of America

## Abstract

Recently, Mendes et al. [1] described the use of a liquid tool (water) in captive orangutans. Here, we tested chimpanzees and gorillas for the first time with the same “floating peanut task.” None of the subjects solved the task. In order to better understand the cognitive demands of the task, we further tested other populations of chimpanzees and orangutans with the variation of the peanut initially floating or not. Twenty percent of the chimpanzees but none of the orangutans were successful. Additional controls revealed that successful subjects added water only if it was necessary to obtain the nut. Another experiment was conducted to investigate the reason for the differences in performance between the unsuccessful (Experiment 1) and the successful (Experiment 2) chimpanzee populations. We found suggestive evidence for the view that functional fixedness might have impaired the chimpanzees' strategies in the first experiment. Finally, we tested how human children of different age classes perform in an analogous experimental setting. Within the oldest group (8 years), 58 percent of the children solved the problem, whereas in the youngest group (4 years), only 8 percent were able to find the solution.

## Introduction

A variety of sophisticated tool-using behavior is known to occur in several vertebrates, including birds and mammals [2]–[4]. Reports of such behavior originate from natural observations [5]–[8] as well as from experimental studies [9]–[12]. The vast majority of tools used by animals consists of solid materials or are constructed from them.

Recently, Mendes, Hanus and Call [1] reported five orangutans repeatedly spitting water into a tube to retrieve a peanut that was floating at the bottom of the tube in a small amount of water. Releasing water from their mouths into the tube raised the water level and brought the peanut within reach. Additionally, control conditions demonstrated that spitting inside the tube was not a general response that subjects displayed upon encountering an out-of-reach reward. In particular, orangutans did not spit water into an empty tube upon encountering a peanut that was out of reach (in front of the tube). These data suggested that their spitting was goal-directed and performed to remove the peanut from the tube.

Even though archerfish (*Toxotes jaculatrix*) are also known to spit water streams to catch their prey [13], most of the spitting behavior seems to be hard-wired, with only some details being amendable to change (i.e., timing and/or direction of spits; [14]. There is no reason to assume that much more cognitive flexibility is involved; for example, it has never been reported that archerfish are capable of using their “spitting behavior” in a completely different and new context. For orangutans on the other hand, water spitting of this sort is not known to be a natural, species-typical behavior, nor did it play any role in the special living conditions of that particular zoo population tested by Mendes and colleagues [1].

Furthermore, two elements suggest that this was a manifestation of insightful behavior [9], [15]: First, the sudden appearance of spitting into the tube after a period of unsuccessful attempts which did not involve spitting in any way and second, the immediate appearance of spitting when needed without reverting to previous unsuccessful behavior. Although the idea of insight has been criticized because prior experiences may have played a role in the solution, Köhler himself [9], [16] recognized that experience with objects preceded their insightful use. It is very likely that orangutans had multiple experiences with liquids in their mouths and even spat them at objects or other individuals. Moreover, orangutans were familiar with shelled peanuts and they might have even seen them floating in water. It is very likely that those experiences were instrumental in allowing subjects to solve the floating peanut task. However, the information gathered from those experiences still had to be cognitively reorganized/re-used to solve a problem that they had never faced before: a peanut at the bottom of a tube.

Nevertheless, orangutans using water to get a peanut from the bottom of a tube is a phenomenon that deserves further examination. From a comparative point of view, it is unknown whether other species of great apes would be able to solve the task. This information is crucial to making inferences about the evolution of cognitive flexibility in nonhuman primates and humans. It is also important to test other ape populations of the same species to see how widespread this ability is among other individuals within the species.

From a cognitive point of view, it is unclear whether apes would have also solved the task if the peanut had not already been floating in the water. It would seem that, encountering a dry tube with a peanut at the bottom is a more demanding task than encountering one with a floating peanut, because it requires thinking about water as a possible solution without having already seen it or its effect. Another aspect that requires further scrutiny is whether or not apes might have a general tendency to add water to the tube regardless of the presence of the reward. Although Mendes et al. [1] ran a series of control conditions to assess whether orangutans spat water indiscriminately into the tube regardless of the position of the peanut, more data would contribute to confirming their results. The aim of our study was to provide some answers to the open questions raised by Mendes et al. 's [1] results.

In Experiment 1, we tested chimpanzees and gorillas housed in the same facility as the orangutans tested by Mendes et al. [1], and used the same method. In Experiment 2, we expanded our sample by including two new populations of chimpanzees and orangutans living in sanctuaries in Uganda and Indonesia, respectively. In addition to the original test condition in which the peanut was floating in a small amount of water, we presented a condition with the peanut lying at the bottom of a completely dry tube. Furthermore, successful subjects were presented with a series of control conditions to investigate whether or not subjects added water only when it was required for solving the task.

Experiment 3 tested the hypothesis that functional fixedness [17], [18] may have been responsible for the difference in performance between the two chimpanzee populations tested in Experiment 1 (Leipzig) and Experiment 2 (Ngamba). Functional fixedness involves cognitive limitations for using a tool (here actually the mechanical source of the tool—namely the water dispenser) in an unusual way [19]. We conjectured that Leipzig chimpanzees might have failed to use water from the drinking devices installed in their quarters to solve the task because they mainly associated those devices with drinking to satiate thirst (which was not the case for the Ngamba chimpanzees). We tested this idea by installing a new water dispenser (‘dispenser’ from now on) and retesting some of the chimpanzees in the floating peanut task. There are some observations in the literature that may qualify as functional fixedness (e.g., [9]) and it has been recently discussed as a potential factor influencing cognitive performance of elephants [20]. However, to our knowledge, this phenomenon has not been systematically investigated in nonhuman animals so far.

In Experiment 4, we tested the ability of 4-, 6-, and 8-year-old children to solve the floating peanut task in an experimental setting analogous to that presented to the apes. We recruited relatively older children because the demanding task requires a great deal of innovation and creativity. Like the apes in Experiment 2, half of the children received the condition in which the tube was quarter filled with water and half of them received the condition in which the tube was empty.

## Experiment 1: Leipzig Chimpanzees and Gorillas

The goal of this experiment was to investigate and compare the performances of chimpanzees and western lowland gorillas with those of the orangutans tested in Mendes et al. 's study [1]. As in the original experiment, the task required subjects to retrieve a peanut from inside a Plexiglas tube by collecting water from a dispenser and then spitting it into the tube in order to make the peanut float and bring it within the subject's reach.

### Method

#### Subjects

Twenty-four subjects participated in the present study, 19 chimpanzees and 5 gorillas (see Table 1 for the details). The chimpanzee group consisted of 5 males (*M*_age_  = 12.4 years, age range: 4–30 years) and 14 females (*M*_age_  = 17.9 years, age range: 6–31 years); the gorilla group consisted of 1 male (25 years) and 4 females (*M*_age_  = 19.0 years, age range: 9–29 years). All of them were socially housed at the Wolfgang Köhler Primate Research Center (WKPRC) located in the Leipzig Zoo, Germany. Although subjects had received a variety of cognitive tests during the last 8 years (see http://wkprc.eva.mpg.de for additional details), this was the first time that they were confronted with the floating peanut task or any other task that entailed extracting food from the bottom of a vertically oriented tube. Nevertheless, some subjects had been confronted with tasks in which they had to extract a reward from a horizontally-oriented tube such as the trap tube task [21], [22].

**Table 1:** Overview of test participation for each ape population.

Subject	Species	Age	Sex	Location	Rearing history	Participation
Alex	*Pan troglodytes*	5y	M	WKPRC	Hand reared	Exp1 Exp3
Alexandra	*Pan troglodytes*	6y	F	WKPRC	Hand reared	Exp1 Exp3
Annett	*Pan troglodytes*	6y	F	WKPRC	Hand reared	Exp1 Exp3
Corry	*Pan troglodytes*	29y	F	WKPRC	Hand reared	Exp1 Exp3
Dorien	*Pan troglodytes*	25y	F	WKPRC	Hand reared	Exp1 Exp3
Fifi	*Pan troglodytes*	12y	F	WKPRC	Mother	Exp1 Exp3
Fraukje	*Pan troglodytes*	31y	F	WKPRC	Hand reared	Exp1 Exp3
Frodo	*Pan troglodytes*	13y	M	WKPRC	Mother	Exp1
Gertruida	*Pan troglodytes*	12y	F	WKPRC	Mother	Exp1 Exp3
Jahaga	*Pan troglodytes*	13y	F	WKPRC	Mother	Exp1 Exp3
Lobo	*Pan troglodytes*	3y	M	WKPRC	Mother	Exp3
Lome	*Pan troglodytes*	4y	M	WKPRC	Mother	Exp1 Exp3
Natascha	*Pan troglodytes*	27y	F	WKPRC	Hand reared	Exp1
Patrick	*Pan troglodytes*	10y	M	WKPRC	Mother	Exp1 Exp3
Pia	*Pan troglodytes*	7y	F	WKPRC	Mother	Exp1 Exp3
Riet	*Pan troglodytes*	29y	F	WKPRC	Hand reared	Exp1 Exp3
Robert	*Pan troglodytes*	30y	M	WKPRC	Hand reared	Exp1
Sandra	*Pan troglodytes*	14y	F	WKPRC	Mother	Exp1 Exp3
Swela	*Pan troglodytes*	11y	F	WKPRC	Mother	Exp1 Exp3
Tai	*Pan troglodytes*	5y	F	WKPRC	Mother	Exp3
Ulla	*Pan troglodytes*	29y	F	WKPRC	Hand reared	Exp1 Exp3
Unyoro	*Pan troglodytes*	10y	M	WKPRC	Mother	Exp3
Bebe	*Gorilla gorilla*	27y	F	WKPRC	Mother/Hand reared	Exp1
Gorgo	*Gorilla gorilla*	25y	M	WKPRC	Hand reared	Exp1
N'Diki	*Gorilla gorilla*	29y	F	WKPRC	Mother/Hand reared	Exp1
Ruby	*Gorilla gorilla*	9y	F	WKPRC	Hand reared	Exp1
Viringika	*Gorilla gorilla*	11y	F	WKPRC	Mother	Exp1
Asega	*Pan troglodytes*	7y	M	NICS	Mother/Hand reared	Exp2
Bahati	*Pan troglodytes*	15y	F	NICS	Mother/Hand reared	Exp2
Baluku	*Pan troglodytes*	7y	M	NICS	Mother/Hand reared	Exp2
Becky	*Pan troglodytes*	16y	F	NICS	Mother/Hand reared	Exp2
Bili	*Pan troglodytes*	7y	F	NICS	Mother/Hand reared	Exp2
Bwambale	*Pan troglodytes*	6y	M	NICS	Mother/Hand reared	Exp2
Connie	*Pan troglodytes*	26y	F	NICS	Mother/Hand reared	Exp2
Ikuru	*Pan troglodytes*	10y	F	NICS	Mother/Hand reared	Exp2
Indi	*Pan troglodytes*	9y	M	NICS	Mother/Hand reared	Exp2
Kalema	*Pan troglodytes*	9y	M	NICS	Mother/Hand reared	Exp2
Katie	*Pan troglodytes*	18y	F	NICS	Mother/Hand reared	Exp2
Kidogo	*Pan troglodytes*	21y	F	NICS	Mother/Hand reared	Exp2
Kisembo	*Pan troglodytes*	6y	M	NICS	Mother/Hand reared	Exp2
Nakuu	*Pan troglodytes*	4y	F	NICS	Mother/Hand reared	Exp2
Namukiza	*Pan troglodytes*	6y	F	NICS	Mother/Hand reared	Exp2
Nani	*Pan troglodytes*	4y	F	NICS	Mother/Hand reared	Exp2
Natasha	*Pan troglodytes*	15y	F	NICS	Mother/Hand reared	Exp2
Nkumwa	*Pan troglodytes*	9y	F	NICS	Mother/Hand reared	Exp2
Okech	*Pan troglodytes*	4y	M	NICS	Mother/Hand reared	Exp2
Pasa	*Pan troglodytes*	6y	F	NICS	Mother/Hand reared	Exp2
Sally	*Pan troglodytes*	14y	F	NICS	Mother/Hand reared	Exp2
Sophie	*Pan troglodytes*	19y	F	NICS	Mother/Hand reared	Exp2
Sunday	*Pan troglodytes*	18y	M	NICS	Mother/Hand reared	Exp2
Umutama	*Pan troglodytes*	9y	M	NICS	Mother/Hand reared	Exp2
Yoyo	*Pan troglodytes*	6y	F	NICS	Mother/Hand reared	Exp2
Bono	*Pongo pygmaeus*	7y	M	OFI	Mother/Hand reared	Exp2
Dego	*Pongo pygmaeus*	6y	M	OFI	Mother/Hand reared	Exp2
Isabella	*Pongo pygmaeus*	6y	F	OFI	Mother/Hand reared	Exp2
Janu	*Pongo pygmaeus*	6y	M	OFI	Mother/Hand reared	Exp2
Jecky	*Pongo pygmaeus*	6y	M	OFI	Mother/Hand reared	Exp2
Jidan	*Pongo pygmaeus*	6y	M	OFI	Mother/Hand reared	Exp2
Lori	*Pongo pygmaeus*	6y	F	OFI	Mother/Hand reared	Exp2
Paiton	*Pongo pygmaeus*	6y	M	OFI	Mother/Hand reared	Exp2
Puji	*Pongo pygmaeus*	6y	F	OFI	Mother/Hand reared	Exp2

Age in years; F =  female, M =  male; WKPRC  =  Wolfgang Köhler Primate Research Center; OFI  =  Orangutan Foundation International, Indonesia; NICS  =  Ngamba Island Chimpanzee Sanctuary, Uganda.

Research at the WKPRC was performed in accordance with the recommendations of the Weatherall report “The use of non-human primates in research”. Groups of apes were housed in semi-natural indoor and outdoor enclosures with regular feedings, daily enrichment and water ad lib. Subjects voluntarily participated in the study and were never food or water deprived. Research was conducted in the sleeping and/or observation rooms. No medical, toxicological or neurobiological research of any kind is conducted at the WKPRC. Research was non-invasive and strictly adhered to the legal requirements of Germany. The study was ethically approved by an internal committee at the Max Planck Institute for Evolutionary Anthropology. Animal husbandry and research comply with the “EAZA Minimum Standards for the Accommodation and Care of Animals in Zoos and Aquaria”, the “WAZA Ethical Guidelines for the Conduct of Research on Animals by Zoos and Aquariums” and the “Guidelines for the Treatment of Animals in Behavioral Research and Teaching” of the Association for the Study of Animal Behavior (ASAB). IRB approval was not necessary because no special permission for the use of animals in purely behavioral or observational studies is required in Germany.

#### Apparatus and Procedure

The apparatus and procedure were the same as in the Mendes et al. [1] study. A transparent Plexiglas tube (26 cm long, 5 cm wide) was vertically attached to a panel inside the subjects' testing room. The bottom end of the tube was closed and the top was open; three metal rings held the tube in place. The tube was quarter filled with water and a shelled peanut floated inside the tube, unreachable for the subjects. A dispenser that was situated 0.5–1 m from the tube has always been in the testing room since its construction, and thus subjects were familiar with its presence and its use. Prior to a subject's entrance, the testing room was cleared of any material that could potentially be used as a tool to reach the peanut. There was no visual contact between the tested subject and other conspecifics.

**Figure 1:**
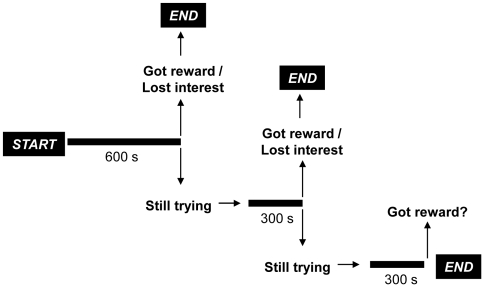
Schematic illustration of the procedure used in Experiment 1and 2.

Each subject received a total of eight trials (one trial per day). Each trial had a maximum duration of 20 minutes. The first 10 minutes were standard, meaning that all of the subjects received that exposure time regardless of their motivation or effort. The trial ended if the subjects retrieved the reward earlier. If the subject was still working to get the peanut after 10 minutes, the experimenter (E) allowed an additional 5-minute period. Again, if the subject retrieved the reward or lost interest, the trial was terminated but if the subject remained interested in the task, it continued for additional 5 minutes. Consequently, each subject had a maximum of 20 minutes per trial to solve the problem and obtain the reward, provided that they showed continued interest during the trial (see Figure 1). E provided no specific cues on how to solve the task and was only allowed to knock on the tube or call the subject's name in order to gain its attention.

#### Data scoring and analyses

We scored the frequency of chimpanzees' spitting behavior, as well as whether or not subjects were ultimately successful, plus the time the subjects were generally interested in the task.

### Results

None of the 5 gorillas and 19 chimpanzees retrieved the peanut from inside the tube. Additionally, none of the subjects added any water to the tube; even though gorillas spent on average about 7 minutes (*M* = 7.34, *SD* = 2.55) and chimpanzees about 10 minutes (*M* = 10.63, *SD* = 0.74) actively trying to get the reward.

### Discussion

The solution to this task required subjects to take water from the dispenser and spit it into the tube in order to raise the water level and bring the peanut within reach. None of the subjects was able to find the appropriate solution to the task. We doubt that lack of motivation accounts for this failure. Subjects appeared interested and behaved actively in trying to extract the peanut from the tube. The majority tried different—though unsuccessful—strategies: hand actions such as pulling, lifting, banging, or inserting their fingers, and mouth actions such as biting and licking. Some subjects even collected water from the dispenser and spat it at E, but never into or at the tube. Only some chimpanzees showed this behavior that could be interpreted as frustration at their failure to get the peanut.

The discrepancy between the chimpanzees' and gorillas' performance in the current study and the orangutans' in the Mendes et al. study [1] is striking. After eight trials, none of the apes in the current study added water to the tube, whereas all five orangutans solved the task from the first trial onwards. It should be stressed that all three ape species were housed under the same conditions at the same facility (WKPRC, Leipzig), and the apparatus and the procedure were identical for all apes. Given that chimpanzees are thought to be especially skillful and innovative problem solvers (e.g., [9], [23]), the current findings are all the more puzzling. The comparatively small sample size involved in the reported studies may have contributed to these discrepant results. In particular, it is unclear whether the observed differences between orangutans on one side and chimpanzees and gorillas on the other side reflect a genuine interspecific difference in problem-solving abilities or whether they represent a mere sampling artefact. In the next experiment, we took up this question by testing other samples of orangutans and chimpanzees on the floating peanut task.

Another outstanding issue in the original Mendes at al. study [1] is whether or not the presence of water inside the tube influenced the orangutans' behavior. In other words, how crucial is seeing a floating peanut to solving the task? Although Mendes et al. [1] included control conditions that addressed this issue by using an empty tube, these conditions were conducted after the experimental condition. Once subjects had solved the problem with the floating peanut, they also succeeded when the tube was dry, which suggests that seeing water was not necessary for producing a solution or else it could have been due to a carry-over effect of the earlier study. And so, it is unclear whether subjects would be able to solve the task without initially seeing any water inside the tube. We addressed this issue in the next experiment.

## Experiment 2: Sanctuary Orangutans and Chimpanzees

The first goal of this experiment was to test one additional sample of sanctuary-housed chimpanzees and orangutans to confirm the observed differences between the chimpanzees and orangutans housed in Leipzig. The second goal of this experiment was to investigate whether apes were able to solve the task when seeing the peanut at the bottom of a dry tube rather than floating in water. Half of the subjects received the original test version with a quarter-filled tube and a floating peanut, whereas the other half was confronted with a dry tube and a peanut lying at its bottom.

### Method

#### Subjects

Thirty-five subjects participated in the present study (see Table 1 for the details): Ten orangutans housed at the Orangutan Care Center Pasir Panjang in Kalimantan, Indonesia and 25 chimpanzees housed at the Ngamba Island Chimpanzee Sanctuary, Uganda. The orangutan group consisted of 6 males (*M*_age_  = 6.2 years, age range: 6–7 years) and 4 females (*M*_age_  = 5.3 years, age range: 3–6 years); the chimpanzee group consisted of 9 males (*M*_age_  = 8.3 years, age range: 4–18 years) and 16 females (*M*_age_  = 12.3 years, age range: 4–26 years). Subjects in both sanctuaries were individually tested and were not deprived of food or water during the experiment.

Research at Ngamba Island and Pasir Panjang was performed in accordance with the recommendations of the Weatherall report “The use of non-human primates in research”. All subjects were allowed to spend several hours per day in surrounding tropical rain forest, received regular feedings and water ad lib. Subjects voluntarily participated in the study and were never food or water deprived.

No medical, toxicological or neurobiological research of any kind is conducted at neither of the sanctuaries. Research was non-invasive, strictly adhered to the legal requirements of Uganda and Indonesia and was approved and reviewed by the Ugandan Wildlife Authorities (UWA) and the Ugandan National Council for Science and Technology (UNCST) as well as the Indonesian Institute of Sciences (LIPI). The study was ethically approved by committees of the Max Planck Institute for Evolutionary Anthropology and the two sanctuaries involved (Chimpanzee Sanctuary & Wildlife Conservation Trust, Orangutan Care Center Pasir Panjang). Animal husbandry and research comply with the “PASA Primate Veterinary Healthcare Manual” and the “Guidelines for the Treatment of Animals in Behavioral Research and Teaching” of the Association for the Study of Animal Behavior (ASAB).

#### Apparatus and Procedure

We used the same apparatus as in Experiment 1. Again, a Plexiglas tube and a dispenser were installed in the testing room, located 0.5–1 meters apart from each other. Subjects received 8 (chimpanzees) and 10 trials (orangutans), depending on specific time constraints at each sanctuary. We conducted two trials per day (morning and afternoon), which again lasted 10–20 minutes (or less if the subject obtained the reward earlier). The procedure was identical to the one used in Experiment 1 (see Figure 1). The dispenser providing water was installed a few days before commencement of the experiment in both sanctuaries. Whereas the dispenser at Ngamba only released water when being pressed, the water in Kalimantan was running all the time (due to technical constraints). The dispenser at the Ngamba was very similar to the one at the WKPRC described in Experiment 1. Besides the described dispenser no other water sources were available.

#### Experimental Phase

There were two conditions: wet and dry. Half of the subjects (5 orangutans, 12 chimpanzees) were presented with the *wet condition* in which a shelled peanut floated inside the tube, as in Experiment 1. Again, the tube was only quarter filled with water so that the peanut could not be reached directly. No other tools were available. The other half of the subjects (5 orangutans, 13 chimpanzees) received the *dry condition* in which there was no water in the tube and the peanut was lying at the bottom of it. The procedure remained exactly the same as in the previous experiment. However, there were additional manipulations at Ngamba: If the subjects presented with the wet condition failed to add any water during the first four trials, they received two additional trials in which the amount of water inside the tube was doubled—although the peanut remained out of direct reach. If subjects presented with the dry condition did not succeed in the first four trials, they immediately received the wet condition from the fifth trial onwards. If those subjects still failed in the following two wet trials, they received two additional wet trials in which the amount of water inside the tube was doubled (e.g., Trial 1–4: dry  = > Trial 5–6: quarter-filled  = > Trial 7–8: half-filled). We presented the additional wet trials to evaluate whether this extra information would facilitate their inventiveness. In all wet-conditions (1/4 and 1/2 full) tubes were filled with water out of subject's view.

#### Control Phase

Upon completing the test phase, the successful subjects advanced to the control phase. Those subjects received three kinds of control trials (top, table, and dry), with each condition occurring four times in total. The order of presentation of the 12 control trials was counterbalanced within and between blocks. In the *top control*, the peanut was attached (glued) to the top of the empty tube and was therefore easily within reach of the subject. In the *table control*, the peanut rested on a platform 30 cm in front of the empty tube beyond the subject's reach. The *dry control* was identical to the experimental dry condition (with a peanut located at the bottom of the tube)—therefore representing the only control condition in which water spitting is an appropriate strategy to obtain the reward.

#### Data scoring and analyses

In the experimental phase we scored whether or not subjects spat into the tube and when they were ultimately successful. Additionally, we coded other tube-directed behavior that was performed with hands and/or feet (e.g., pulling, pushing, lifting). We examined further if subjects behaved differently on those variables before and after the solution was discovered. We calculated the medians (*mdn*) and ranges for spitting latencies and tube-directed actions. In the control phase we scored how often subjects spat water into the tube during the first 2 minutes of each condition and calculated the mean percentage of trials in which subjects spat into the tube at least once. In addition, we calculated the mean latency to the first spit and until they obtained the reward in the dry control.

We used non-parametric statistics because the data did not meet the homogeneity of variance supposition. The Friedman exact test was used to compare the percentage of trials in which spitting occurred across all three control conditions. Wilcoxon exact tests for related samples were used to conduct pair-wise comparisons between the conditions. We used the Mann-Whitney exact test to investigate the effect of sex and a Spearman correlation to investigate the effect of age on the percentage of trials in which subjects spat into the tube. For Wilcoxon tests and for Mann-Whitney test the effect sizes (estimate *r*) are reported. Estimate *r* can be interpreted as correlation coefficient [24]. All statistical tests were two-tailed.

### Results

#### Experimental phase

None of the 10 orangutans solved the task. Only two subjects (one 6 year old male, one 6 year old female) spat water into the tube, but failed to continue doing so to the point where they could have reached the reward. These two subjects belonged to the dry-condition group, whereas none of the subjects from the wet-condition group used the water to spit.

Five of 24 chimpanzees solved the task. Two of them (one 6 year old female, one 4 year old male) belonged to the dry-condition group, and three of them (one seven year old and one 18 year old female, one 9 year old male) belonged to the wet-condition group. There was no significant difference between the wet and the dry condition with respect to the number of successful subjects (Fisher's exact test: *p* = 1.0). Four subjects found the solution within the first trial and one subject in the second trial. The five successful subjects added water on average in 73.5 percent of the trials and got the peanut on average in 65.5 percent of the trials.

It took the successful chimpanzees on average 232 seconds (*mdn*) to produce the very first spit (*latency*_DRY_ range  = 167–232; *latency*_WET_ range  = 5–533) and 578 seconds (*mdn*) to get the reward for the first time (*latency*_DRY_ range  = 520–811; *latency*_WET_ range  = 459–618).

Due to individual differences in their spitting techniques, their facial anatomy, and the test condition, subjects needed between 2 and 12 spits to bring the peanut within reach. However, once the solution was discovered, subjects spat much more readily during the following trials; now it took them on average only 41 seconds (*mdn*) to produce the first spit (*latency*_DRY_ range  = 17–35; *latency*_WET_ range  = 46–54) and 131 seconds (*mdn*) to get the reward (*latency*_DRY_ range  = 65–242; *latency*_WET_ range  = 85–177). At the same time, the frequency of tube-directed hand and foot actions declined dramatically from an average of 25.5 (*mdn*) prior to finding the solution (*action*_DRY_ range  = 10.9–58.0; *action*_WET_ range  = 1.0–40.0) to an average of 0.9 (*mdn*) after solving the task (*action*_DRY_ range  = 0–1.6; *action*_WET_ range  = 0.3–1.5). For spitting latency, three out of four subjects showed the reported ‘before-after-decline’. For hand/foot actions, all four subjects showed the reported ‘before-after-decline’. Despite the obvious drop-down of spitting latency and hand/foot actions both ‘before-after comparisons’ failed to reach statistical significance (Wilcoxon exact test for spitting latency: *T^+^* = 3, *n* = 4, *p* = .250; effect size *r_estimate_*  = −.73; Wilcoxon exact test for hand/foot actions: *T^+^* = 3, *n* = 4, *p* = .125; effect size *r_estimate_*  = −.91)—most probably because of the small sample size (four successful subjects) and the big variances involved. Nevertheless, we believe that the reported decline reflect that ineffective manual manipulations (e.g., hand and/or foot actions) were replaced by spitting once the solution was found.

The peculiar behavior of two chimpanzees is worth mentioning: One adult female chimpanzee was successful during her first trial and continued to spit water into the tube for one more trial (but without getting the reward). She finally stopped spitting entirely from the third trial onwards. One juvenile male chimpanzee (dry-condition group) solved the problem during the first trial but failed to add enough water to reach the peanut in the following trials. He spat water during two more trials but had severe difficulties in channeling the water into the small opening of the tube and finally lost interest after several (4) unsuccessful attempts. We decided to present him with the wet condition but he still did not manage to add enough water during the first two trials,. However, when the amount of water inside the tube was increased (to half filled) he finally spat enough water (to reach the peanut) and continued to solve the problem from the third trial onwards throughout the five remaining trials. It seemed that even though he had already found the solution during the very first trial, he simply failed to master the appropriate spitting technique, which caused him to give up. Because less water is needed to solve the task in the wet condition, he was once again motivated and succeeded up to the end of the experiment.

In addition to the five successful subjects, four other individuals spat water into the tube but failed to complete the task; in other words, they did not add enough water to bring the peanut within reach. One of them (a male) belonged to the dry-condition group, and three of them (two males, one female) belonged to the wet-condition group. On average, those four unsuccessful subjects spat water (at least once) in 41.8 percent of all trials.

Finally, no sex differences concerning the overall spitting frequency could be identified, either for chimpanzees (Mann-Whitney exact test: *U* = 51, *n*_male_  = 9, *n*_female_  = 16, *p* = .172; effect size *r_estimate_*  = −.28) or orangutans (Mann-Whitney exact test: *U* = 10, *n*_male_  = 6, *n*_female_  = 4, *p* = 1.0; effect size *r_estimate_*  = .26). Furthermore, there was no correlation between age and subjects' spitting frequency for either of the two species (*r*_chimp_(25)  = −.201, *p* = .335, *r*_orang_(10) <.001, *p* = 1.0). We obtained similar results for correlations between age and success (*r*_chimp_(25)  = −.197, *p* = .344).

#### Control Phase

Although only 4 chimpanzees consistently solved the task during the test phase (recall that Katie initially solved the task but then lost interest), a total of 10 subjects entered the control phase. To increase our sample size, we included 6 additional chimpanzees from Ngamba who originally failed the task but mastered it in the course of an observational learning experiment [25]. All of these 6 subjects re-invented the solution after they had seen successful demonstrations (via emulation learning).

**Figure 2:**
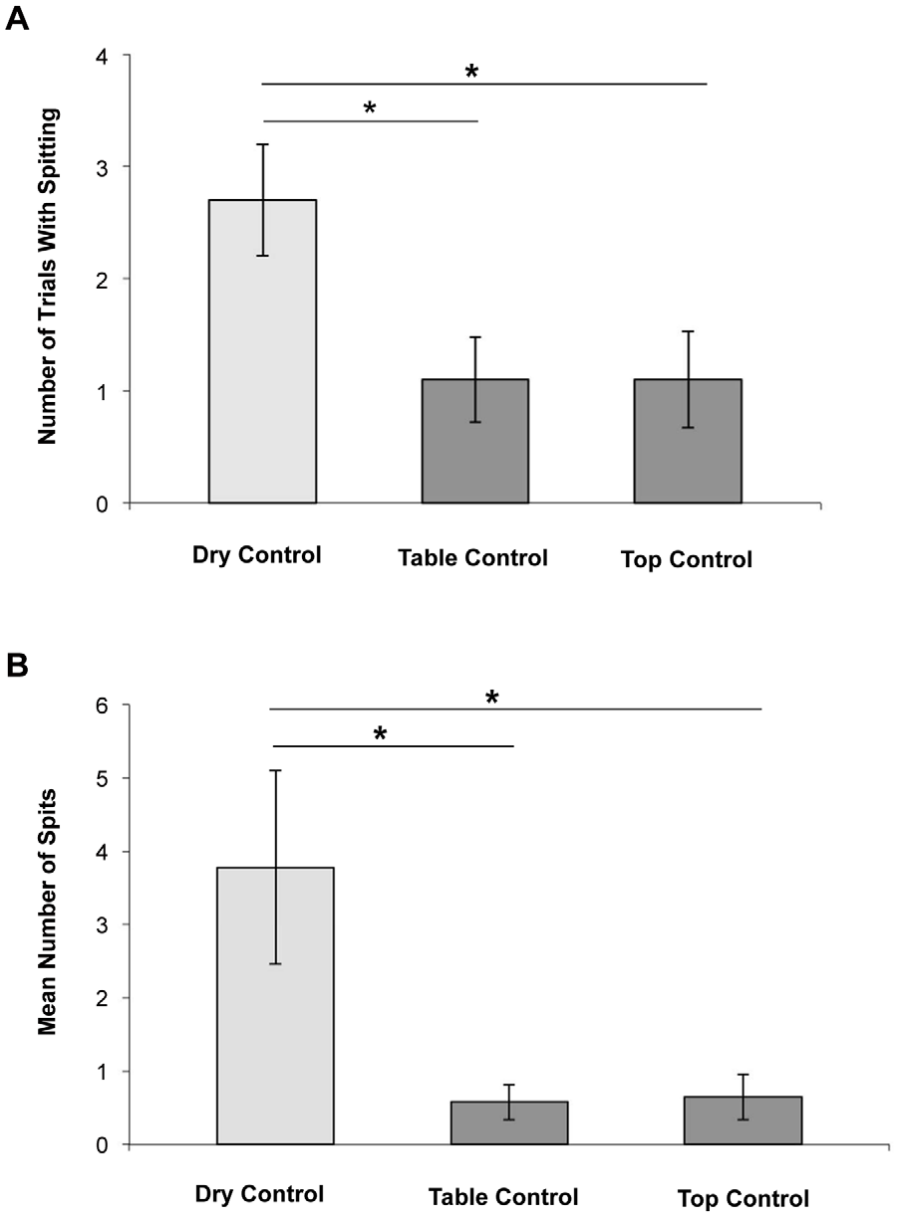
Spitting behavior for each of the three control conditions in Experiment 2. 2a) Mean number of trials in which subjects spat into the tube; 2b) Mean number of water spits that subjects added in total. * p<.05. Error bars depict the standard errors of the means.

There was a significant difference between the three control conditions in the percentage of trials in which spitting occurred at least once (Friedman exact test: *F* = 16.76, *n* = 10, *p*<0.001). Pair-wise comparisons revealed that chimpanzees added water significantly more often in the dry control than in the top control (Wilcoxon exact test: *T^+^* = 9, *n* = 10, *p* = .004; effect size *r_estimate_*  = −.86) and the table control (Wilcoxon exact test: *T^+^* = 9, *n* = 10, *p* = .004; effect size *r_estimate_*  = −.86)—which suggests that the willingness to spit into the tube increased only when adding water is physically required to obtain the reward. In contrast, there was no significant difference between the top control and the table control with respect to the number of trials in which spitting occurred (Wilcoxon exact test: *T^+^* = 1, *n* = 10, *p* = 1.00; effect size *r* = 0; see Figure 2a).

An analysis of the average spitting frequency confirmed a significant difference between the three control conditions (Friedman test: *F* = 11.03, *n* = 10, *p* = .002). Pair-wise comparisons revealed that chimpanzees spat significantly more often in dry control trials compared to top control (Wilcoxon exact test: *T^+^* = 8, *n* = 10, *p* = .008; effect size *r_estimate_*  = −.80) and the table control trials (Wilcoxon exact test: *T^+^* = 8, *n* = 10, *p* = .008; effect size *r_estimate_*  = −.81). In contrast, there was no difference between the top control and the table control concerning the mean number of spits produced by the subjects (Wilcoxon exact test: *T^+^*  = 3, *n* = 10, *p* = 1.00; effect size *r_estimate_*  = −.04). Subjects spat water about seven times more often in the dry control compared to the two other control conditions (see Figure 2b).

Finally, we analyzed when the initial spitting occurred during trials in which water was used. There was again a significant difference between conditions in the latency until the first spit occurred (Friedman exact test: *F* = 6.40, *n* = 5, *p* = .039) Although subjects tended to add water earlier in the dry control than in the other control conditions (*M*_latency-DRY-Control_  = 20.7 s, *M*_latency-TOP-Control_  = 64.5 s, *M*_latency-TABLE-Control_  = 41.0 s), pair-wise comparisons failed to reach significance level (Wilcoxon exact tests: dry vs. top: *T^+^*  = 5, *n*  = 5, *p* = .063; effect size *r_estimate_*  = −.91; dry vs. table: *T^+^*  = 5, *n* = 5, *p* = .094; effect size *r_estimate_*  = −.80; top vs. table: *T^+^*  = 5, *n* = 5, *p* = .125; effect size *r_estimate_*  = −.79).

### Discussion

One fifth of the orangutans (n = 2) added water to the tube but did not add enough water to get the peanut. In contrast, more than a third of the chimpanzees (n = 9) added water to the tube, five of whom added enough water to get the peanut. Four of those chimpanzees continued to solve the task in subsequent trials. According to the latency data (e.g. appearance of the first spit) the extra information of water inside the tube (wet condition) did not seem to stimulate chimpanzees' inventiveness. Furthermore, control tests showed that successful chimpanzees preferentially added water to the tube when the peanut was inside the tube, not simply when the peanut was present yet out of reach. Chimpanzees seemed to add water exclusively to affect the position of the peanut, which confirms the goal-directedness of their behavior. Results are also consistent with the notion of insightful behavior [9], [26]. Next, we discuss in more detail the orangutans' and chimpanzees' results in turn.

The orangutans' negative results stand in stark contrast to the results obtained by Mendes et al. [1]. The main difference in the setup between the two studies was that a running stream of water was visually available in the current study. Arguably, this methodological difference should in fact have favoured the subjects in our study by calling their attention to the water. Interestingly, the two orangutans that spat water into the tube belonged to the dry-condition group, that is, they had not seen the peanut floating inside the tube. It is conceivable that a lack of motivation may have played an important role in the orangutans' failure to solve the task—even though the reward (peanut) was identical in all experiments. The majority of them lost interest in the tube/task after a few unsuccessful attempts, despite repeated efforts by the experimenter to draw their attention to the tube. Perhaps a larger reward would have increased subjects' motivation to continue trying to solve the task.

Unlike the orangutans, the chimpanzees overall seemed much more interested in the task and therefore more motivated to find a solution, which resulted in various strategies to retrieve the peanut (e.g., hand actions such as pulling, lifting, banging, or inserting their fingers, and mouth actions such as biting and licking). Out of the nine chimpanzees that spat water at least once into the tube, five subjects finally added enough water to obtain the reward. It is unclear why the other four subjects stopped spitting water after having made a “first step” towards the final goal. It appeared that all of the subjects who spat unsuccessfully released only tiny amounts of water, preferring to swallow most of the water they retrieved from the dispenser. Why one subject (Katie) that solved the problem during the first trial stopped during the following trials remains unclear.

## Experiment 3: Functional Fixedness

Upon completing Experiment 1 and as part of a different project, Tennie, Call, and Tomasello [25] tested the ability of the initially unsuccessful Leipzig chimpanzees to solve the floating peanut task by observation. This study required training one chimpanzee (Frodo) to solve the task in order to become a demonstrator for some chimpanzee subjects. Over a period of several days, to induce Frodo to use water for the task from his usual and familiar dispenser (‘old dispenser’ from now on), several methods were tried. None of these methods made him use the old dispenser for the task. Yet, Frodo would reliably gather water for the task from diverse other sources: a water bottle lifted to the mesh, running water from a hose, a small receptacle full of water, a water jet rising out of a hole in the direct vicinity (i.e., a few cm) of the old dispenser—and also a “new” dispenser. Similar in working design to the old dispenser, the new dispenser was mounted on a plate of a different color and appearance, and was placed in a different location. Although Frodo successfully gathered water from this new water source for the floating peanut task, he could never be enticed to use water from the old dispenser—that was present in Experiment 1—for that purpose. All attempts to call his attention to it by knocking on the dispenser, pointing to it, or approaching the location where it was installed were unsuccessful.

Frodo's behavior was reminiscent of a phenomenon known as functional fixedness (see also introduction). Frodo seemed cognitively blocked to use the old dispenser in an unusual way. “Unusual” can mean several things. First, cognitive limits like these can stem from subjects' past first-hand experience with an object in different contexts (functional fixedness via individual learning) or, second, from a mismatch between conventional or normative use of an object as compared to current requirements (functional fixedness via cultural learning; see also [27] for a related distinction). Given that apes' cultural learning is limited (for a recent review see [28], [29]; but see [29]) here we are mainly interested in functional fixedness via past individual learning. Such experience based individual learning will have a lot in common with habit formation as well as other related concepts (e.g., “conservatism”; see also General Discussion).

To Frodo, the old dispenser's fixed function—indicated by its location and gained by personal experience—was primarily to supply him with water to quench his thirst (another known function was to provide water for spitting at people). This might have cognitively blocked Frodo from seeing the old dispenser's potentially new function of providing water to solve the floating peanut task. Thus, while water could itself become a tool in our study, for our functional fixedness approach it is the dispenser that we regard as a crucial mechanical source of the tool that provides water for different functions. The functional fixedness hypothesis would thus explain why Frodo readily used new dispensers of different colors, and at different locations. They simply may not have had the same fixed function as the old dispenser. If the functional fixedness hypothesis is true, it might explain the differences observed between the Leipzig chimpanzees (Exp. 1) and the Ngamba chimpanzees (Exp. 2). All subjects at Ngamba had been tested with a new dispenser (9 of 25 subjects added water at least once and 5 of 25 subjects solved the problem), whereas all 19 subjects in Leipzig had been tested with an old dispenser (and here none of the subjects added water at all). We therefore decided to test functional fixedness as a potential reason for the Leipzig chimpanzees' poor performance and investigated whether their performance would improve to compare to that of the Ngamba chimpanzees if they were presented with a new dispenser.

### Method

#### Subjects

We tested the same Leipzig chimpanzees as in Experiment 1, except for Frodo, Robert, and Natascha (see Table 1 for the details). Frodo was excluded because of his special training history (see above), whereas Robert and Natascha were not available for testing during that time. In addition, we tested three previously untested chimpanzees (Unyoro, Lobo, Tai), bringing the total to 19 subjects. The group consisted of 5 males (*M*_age_  = 6.4 years, age range: 3–10 years) and 14 females (*M*_age_  = 16.3 years, age range: 5–31 years). Prior to the current experiment, none of these subjects had solved the task.

#### Apparatus and Procedure

In addition to the old dispenser used in Experiment 1, a new dispenser was installed, so that the subjects had two dispensers to choose from. This new dispenser was functionally identical to the original one but with differences in design. The metal plate on which it was mounted (10 cm×13 cm) was dissimilar in colour and appearance to the old dispenser and a water-hose extended from its back. We tried to maintain equal distances between the apparatus and the old and the new dispenser. However, for some chimpanzees, this was not possible due to the spatial restrictions of their testing rooms. For these seven subjects, the new dispensers were circa 60 cm closer to the apparatus than the old dispensers (90 vs. 150 cm).

Subjects were divided into two groups. Subjects in the *dry group* first (n = 10) received two trials with the peanut lying at the bottom of an empty tube (dry trials), followed by two additional trials with the peanut floating in a quarter-filled tube (wet trials) if they had not solved the dry trials. Subjects in the *wet group* (n = 9) received four wet trials in total (see procedure of Exp 2 for a detailed description of the two conditions). Both groups received only one trial per day. In all other respects, the procedure was identical to the one in Experiment 1. During all trials, subjects had access to the old and the new dispensers, both of which were functional all the time.

#### Data scoring and analyses

As in the previous experiments we scored the frequency of a chimpanzee's spitting into the tube, whether the subjects were ultimately successful or not, and in addition now, the source from which the water was taken (old dispenser, new dispenser, or both). The data were analyzed in the same way as in previous experiments. Finally, we calculated the mean percentage of trials in which subjects spat into the tube and their success rate to compare the data between chimpanzee populations. For each subject, spitting rate was calculated as number of trials with spitting present divided by total number of trials. Success rate was calculated as number of successful trials divided by total number of trials. Nonparametric tests were used because data did not fulfill the homogeneity of variance supposition. Effect sizes (estimate *r*) are reported for Mann-Whitney test.

### Results

In the dry group (n = 10), two subjects spat water into the tube at least once (Fifi 1st trial, Jahaga 2nd trial). However, none of them added enough water to obtain the reward. In the wet group (n = 9), three subjects spat water into the tube (Lome 1st trial, Ulla 4th trial, Tai 4th trial); two of them (Lome, Tai) added enough water to obtain the reward. Whenever subjects took water they always used the new dispenser to spit into the tube. As the combined data from the dry and the wet group show, five out of 19 subjects added water to the tube at least once, and two of them successfully obtained the peanut.

**Figure 3:**
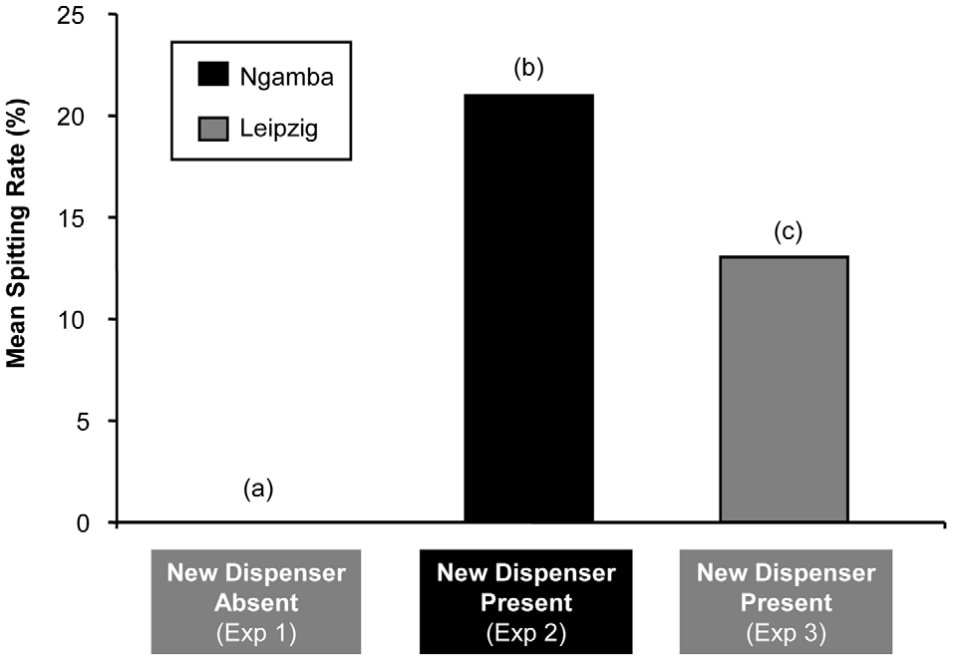
Mean spitting rate for each group  =  Sum of the individual spitting rates divided by number of subjects in each group. (a): 19 subjects from Leipzig tested with the new dispenser absent, (b): 25 subjects from Ngamba tested with the new dispenser present. (c): 16 subjects from (a) plus 3 new subjects from Leipzig tested with the new dispenser present.

These data were compared with those for Experiments 1 and 2. Figure 3 shows the frequency of spitting (regardless of success) for each of the three groups. Chimpanzees that had initial access to a new dispenser in Experiments 2 and 3 spat significantly more often than those that had only access to an “old” (familiar) dispenser in Experiment 1 (Mann-Whitney exact test: *U* = 171, *n*_new_  = 28, *n*_old_  = 19, *p* = .005; effect size *r_estimate_*  = −.42). Also, the previous performance differences between the two populations in Leipzig (Exp 1) and Ngamba (Exp 2) disappeared when the Leipzig chimpanzees were finally given access to a new dispenser (Mann-Whitney exact test: *U* = 212, *n*_Ngamba_  = 25, *n*_Leipzig_  = 19, *p* = .471; effect size *r_estimate_*  = −.01).

As for the success rate, subjects that had initial access to the new dispenser in Experiment 2 and 3 did not perform significantly better than those that had access to the old dispenser only in Experiment 1 (Mann-Whitney exact test: *U* = 209, *n*_new_  = 28, *n*_old_  = 19, *p* = .068; effect size *r_estimate_*  = −.31). Nevertheless, the performance of the Leipzig and Ngamba subjects became more similar when the former were also given access to a new dispenser (Mann-Whitney exact test: *U* = 215, *n*_Ngamba_  = 25, *n*_Leipzig_  = 19, *p* = .452; effect size *r_estimate_*  = −.13).

The three subjects from Leipzig that had initial access to the new dispenser (Exp. 3) are particularly valuable for purposes of comparison with the Ngamba chimpanzees because, unlike the other Leipzig chimpanzees, they faced the test for the first time. There were no significant differences between these two groups in the frequency of spitting (Mann-Whitney exact test: *U* = 34, *n*_Ngamba_  = 25, *n*_Leipzig_  = 3, *p* = .720; effect size *r_estimate_*  = −.06) or success in getting the peanut (Mann-Whitney exact test: *U* = 34, *n*_Ngamba_  = 25, *n*_Leipzig_  = 3, *p* = 0.929; effect size *r_estimate_*  = −.07). 

### Discussion

Adding a new dispenser to the setup increased the frequency of spitting and thereby reduced the differences in performance between the Leipzig chimpanzees in Experiment 1 and the Ngamba chimpanzees in Experiment 2. One possible explanation for our findings is the proposed functional fixedness hypothesis [17], [18].

One could argue that the increased performance observed in this study compared to Experiment 1 was due not to the introduction of the new dispenser but to the retesting of the same chimpanzees. In other words, providing additional trials rather than a new dispenser may explain this result. However, the following reasons make this unlikely: First, the three chimpanzees that received the task for the first time with access to the new dispenser performed at comparable levels to the Ngamba chimpanzees—even though the unequal sample sizes of each group dictates caution in the interpretation. Second, in strict accordance with the functional fixedness hypothesis, subjects in the current experiment gathered the water that they spat into the tube exclusively from the new dispenser and never from the old dispenser. We also observed subjects using the new dispenser as a source of water for drinking as well as for spitting at the experimenter.

The third reason why order effects seem unlikely is that apes either acquired quickly how to spit into the tube or they did not solve it at all. Of the 10 apes (five orangutans from Mendes et al.,[1]; five chimpanzees from Exp. 2) that have solved this task so far independently, nine solved it in the first trial and one in the second. In contrast, none of the original subjects from the Leipzig group solved the problem during the course of eight trials. Fourth, there is the interesting case of Frodo, who was adept at solving the task by gathering water from different sources but could not be induced to use water from the old dispenser. These four aspects offer at the very least suggestive evidence that functional fixedness may have been responsible for the differences detected between the Leipzig chimpanzees (Exp. 1) and the Ngamba chimpanzees (Exp. 2) in the floating peanut task.

## Experiment 4: Children

Experiments 2 and 3 as well as the results of Mendes et al. [1] showed that some chimpanzees and orangutans are able to solve the floating peanut task in a flexible and innovative way. In this experiment, we investigated how 4- to 8-year old children performed in the same task in a comparable experimental setting. We selected 4-year-old children because they have not yet developed the level of executive function implicated in problem solving that eight-year old children have already achieved [30]–[32]. Including six year-olds allowed us to trace more precisely the development in problem solving in the floating peanut task.

### Method

#### Participants

Seventy-two children (36 boys and 36 girls) took part in the experiment. There were three age classes: 4 years (*M*_age_  = 50.5 month, age range: 48–54 month), 6 years (*M*_age_  = 74.4 month, age range: 72–78 month), and 8 years (*M*_age_  = 96.6 month, age range: 93–98 month). In each age class, there were 12 boys and 12 girls assigned to one of two conditions, dry and wet (see Experiment 2 and 3). All children (four and six year olds) were recruited from kindergartens and primary schools (eight year olds) in Leipzig, Germany. The majority of children came from a middle-class white background. Research strictly adhered to the legal requirements of Germany and informed consent, in written form, was obtained from the parents of all children who participated in this study. In addition, the study was ethically approved by an internal committee at the Max Planck Institute for Evolutionary Anthropology.

#### Apparatus and Procedure

The same Plexiglas tube was used as in the previous experiments. Instead of a dispenser, a water-filled pitcher was provided in close proximity to the apparatus. The Plexiglas tube was attached to a vertically oriented wooden board (40×10 cm) that was mounted to a table. As in the ape studies, no other tools were available in the testing room.

All children received only one test trial in total. This was because it proved to be impossible to prevent them from conversing with other people before subsequent trials. In order to get used to the pitcher and the test situation, all children were asked to use the pitcher to water some pot plants in the testing room prior to starting the test trial. At this time, the apparatus (tube) was covered by a blanket. After watering the plants, the child (C) was asked to place the pitcher on the test table (50–80 cm distance from the tube) before leaving the room together with the experimenter (E). After a few minutes, C and E entered the room again, and E explained the problem to C: “Let's play a game. Look, there is a peanut inside the tube. If you can get that peanut, you will win a reward (Kinder Surprise). Unfortunately I cannot help you because I have important paperwork to do.” E then sat down in another part of the room (4–6 m distance from the apparatus), where he/she stayed during the entire testing phase.

A trial lasted a maximum of 8 minutes (or less if C got the peanut sooner). If C did not solve the task after 4 minutes had elapsed, E verbally encouraged C to try whatever solution he/she might have in mind (“If you have an idea, just try!”). No other cues were given by E. Finally, after 8 minutes had elapsed, E asked C one last time whether he/she would like to try something else. If the child had no further ideas, the trial ended. All children were given a reward (toy) at the end, regardless of their success.

#### Data scoring and analyses

As in the previous experiments we scored whether or not participants solved the task. In addition we measured the latency up to when the first water was added as well as when participants got the peanut. Finally, we ran a logistic regression to analyze the effect of sex, age class, and test condition (as covariates) on successful performance (as dependent variable). The reported overall effect size (Nagelkerke *r* ^2^) was based on the model summary of that logistic regression. We used a Mann-Whitney exact test for an age comparison for which the effect sizes (estimate *r*) are reported.

### Results

**Figure 4:**
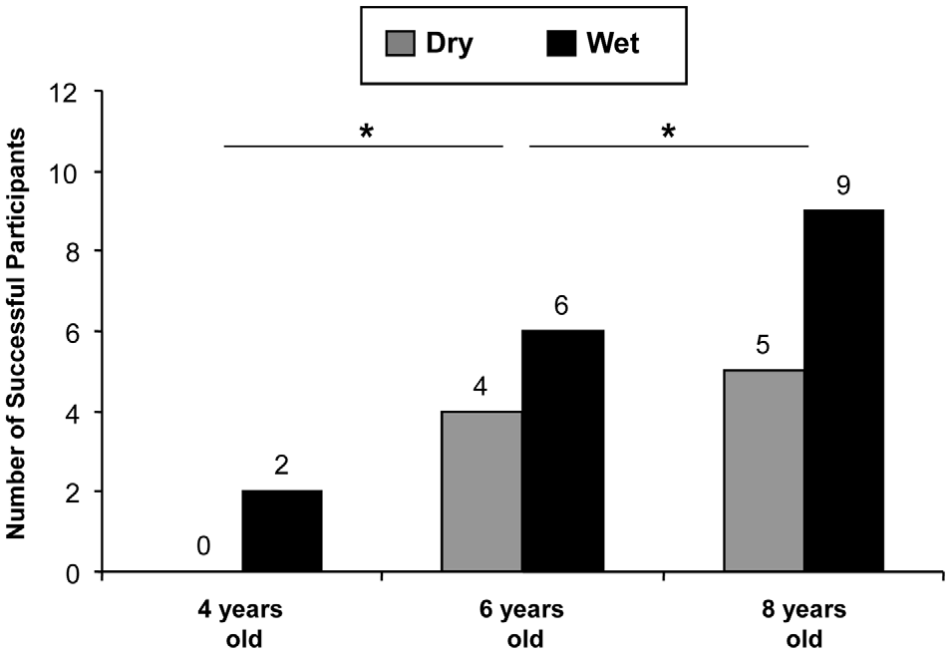
Number of successful children for each of the three age classes. Grey bars represent participants in the dry-condition group; black bars represent participants in the wet-condition group. * p<.05.

Figure 4 presents the number of children who solved the task as a function of age and condition. Within the youngest age class (4 years), only two children solved the task by pouring water into the tube. Those two participants belonged to the wet-condition group. Within the middle age class (6 years), 10 children solved the task: Six of them belonged to the wet-condition group and four belonged to the dry-condition group. Within the oldest age class (8 years), 14 children solved the task: Nine of them belonged to the wet-condition group and five belonged to the dry-condition group. Older children performed significantly better than younger ones (*B* = 1.32, *p* = .01, overall effect size *r* ^2^ = .31). Additionally, participants from the wet group were significantly more successful than those from the dry group (*B* = 1.24, *p* = .03). Gender had no effect on children's ability to solve the task in any of the three age classes (*B*<.001, *p* = 1.00).

Apart from analyzing the success rate, we also identified a clear age effect on the latency until the first portion of water was added into the tube. Due to the small number of successful 4-year-olds, we combined the successful 4- and 6-year-olds (“younger”) and compared them to the successful 8-year-olds (“older”). Older children needed less than half of the time to find the appropriate solution that younger children required (Mann-Whitney exact test: *U* = 40, *n*_young_  = 12, *n*_old_  = 14, *p* = .022; effect size *r_estimate_*  = −.44; *M*_latency_ 4–6-year-olds: 249 s; *M*_latency_ 8-year-olds: 91 s).

### Discussion

Children solved a variant of the floating peanut problem, but success strongly depended on age and condition. Whereas only 8 percent of the 4-year-olds solved the task, this number increased to 42 percent and 58 percent in 6- and 8-year-olds, respectively. Additionally, children who found the peanut floating on water were more likely to solve the task. Taken together, the 8-year-olds who saw the floating peanut were the most successful group (75 percent success), and the 4-year olds who encountered the dry peanut were the least successful ones (0 percent success).

Despite the high success of 8-year-olds in the wet condition, many children in other groups consistently failed to solve the task. We can rule out a motivational deficit in the younger group as an explanation for the results, because they were very interested in the reward and the vast majority of them spent a great deal of time and effort trying to get the peanut. Likewise, we do not assume that the relatively low scores were caused by the children perceiving the water in the pitcher as either unavailable or unusable. Although that possibility cannot be fully excluded, it remains unlikely, given that we explicitly drew the children's attention to the water in the pitcher (watering plants) prior to the test. This in turn raises the possibility that watering the plants may have interfered with solving the task because the pitcher then acquired a “watering function.” However, the children watered the plants only once––which should have been too little exposure to block other functions, making functional fixedness less likely. Moreover, we used a transparent pitcher rather than a typical watering-can to reduce functional fixedness effects as much as possible. Although we could have opted for not having them use the pitcher to water the plants before the task, we felt it important to show them that it was permissible to use the pitcher but without explicitly calling attention to it as a potential tool. Otherwise, the children may have interpreted such behavior as a communicative cue regarding the relevance of the pitcher to the test.

The strategies deployed by each age class in trying to get the peanut were revealing. Younger children tried to solve the problem almost solely by reaching directly towards the peanut with their hands/fingers. They seemed stuck on this particular approach and were unable to readjust their behavior even though it failed completely. The most likely explanation is that it simply did not occur to most of the children to use the water to solve the task. Many of the older children showed greater cognitive flexibility that enabled them to discard the unsuccessful strategy of reaching with their hands or fingers, which they also attempted, and to search for alternative solutions. These children were seemingly capable of enlarging their attentional focus beyond the tube/peanut to other elements present in the room, such as the pitcher of water.

Another important aspect of the children's problem-solving behavior is that they verbalized their failure to solve the task and addressed the experimenter. That is, children in all age classes continually asked the experimenter for help and/or spoke about their inability to solve the problem. Although it was not intended by the experimenter, it seems possible that the children felt a strong social pressure to solve the task. Such social pressure may have suppressed their innovative and exploratory behavior, especially among the younger subjects. Although this problem might have been ameliorated if the experimenter had left the room, leaving the children alone could have had an analogous detrimental effect by making them wary.

### General Discussion

Even though all subjects seemed interested in the reward, neither the chimpanzees nor the gorillas from Leipzig solved the problem in Experiment 1. Subjects from the two sanctuary populations tested in Experiment 2 were more successful: Two out of 10 orangutans added water to the tube but not enough to get the peanut out; nine out of 24 chimpanzees added water to the tube, and five of these got the peanut at the end. In contrast, subjects did not add water to the tube in the control conditions when such an action could not affect the position of the peanut. Experiment 3 showed that introducing a new dispenser to the formerly unsuccessful Leipzig chimpanzees (Exp. 1) eliminated the differences in performance between them and the Ngamba chimpanzees (Exp. 2). Therefore, functional fixedness might explain the difference between the two groups of chimpanzees. Children tested with an analogous setup to that used with the apes also solved the task but their performance varied with age and experimental condition. Four-year-old children failed the task whereas about half of the 6- and 8-year-old children succeeded. Additionally, seeing the peanut floating in the water facilitated the task substantially, but mostly for the older children.

Results from the control conditions (Exp. 2) confirmed Mendes et al. 's [1] findings obtained with orangutans: Successful chimpanzees spat water into the tube mostly when it affected the location of the peanut. These data give credence to the hypothesis that spitting water in the tube was a goal-directed action aimed at getting access to the peanut located inside the tube. More importantly, our results go beyond those of Mendes et al. [1] by showing that several chimpanzees and children older than four years of age were able to solve the problem without initially seeing the peanut floating inside the tube. Seeing the floating peanut facilitated the task for children but not for chimpanzees, although our sample size may have been too small to detect such an effect. Despite these advancements, our data still cannot determine whether subjects had anticipated the precise effect that spitting would have on the peanut before their initial spit. Such anticipation would indicate sophisticated cause-effect knowledge between their actions (i.e., spitting) and their outcomes (i.e., making the peanut accessible). Similarly, we cannot determine whether subjects mentally rehearsed (and discarded) other options besides spitting inside the tube. Future studies are required to address these outstanding questions.

One of the most striking contrasts found in the current study is the difference between different groups of the same species. Initially, Ngamba chimpanzees outperformed Leipzig chimpanzees, but such differences disappeared with the introduction of a new dispenser for the Leipzig chimpanzees. The results of Experiment 3 are consistent with the idea that functional fixedness may have accounted for the initial poor performance of the Leipzig chimpanzees. This would mean that chimpanzee problem solving, like human problem solving, can be affected by functional fixedness. It is thus conceivable that functional fixedness (assumed to be a human universal [33]), can also be found in chimpanzees and probably also in other tool-using taxa (e.g., birds). In contrast, the Leipzig orangutans [1] did not experience the same difficulty and all solved the task in the first trial, which suggests that orangutans did not experience functional fixedness in this task—or were able to overcome it. Why chimpanzees but not orangutans seemed affected by functional fixedness remains an open question. Unexpectedly, none of the sanctuary orangutans solved the task, even though at least two of them spat water into the tube. The failure of the sanctuary orangutans cannot be attributed to functional fixedness because the dispenser system was totally new to them. Gorillas also performed poorly but just like the Leipzig chimpanzees in Experiment 1, it is possible that functional fixedness contributed to this outcome. Future studies with larger samples (and with some methodological modifications; including new dispensers) are required to draw firmer conclusions on gorillas' performance in the floating peanut task.

Functional fixedness may also be related to results recently obtained in observational great ape studies that found that, once a solution was found to a problem, chimpanzees became reluctant to change their strategies—referred to as “conservatism” [34], [35] or linked to the concept of “reduced readiness for change”, which might be plausibly related to this phenomenon [36] (but see also [9] p. 40). Currently it remains unclear whether this phenomenon was truly due to the active conservation of old strategies—or else due to functional fixedness. The difference between these two possibilities lies in a difference of choice: in the first case, subjects may realize alternatives but actively opt against them, whereas in the latter case they fail to detect alternatives in the first place.

Pooling together our current results with those of Mendes et al. [1] show that chimpanzees and orangutans performed better than 4-year-old children and worse than 6- and 8-year-olds. However, caution is required when directly comparing children's and apes' performances in this task, due to the various methodological differences between studies. Apes received multiple trials, whereas children received only one. Given that apes succeeded in the first trial or not at all (with two exceptions: one chimpanzee succeeded in the second trial and another one in the fourth trial), this may not have been such a critical difference. Another difference is that water was visually available to children but not to apes—except for the orangutans (Exp. 2). Yet, visual access to water did not seem to have helped either group much: None of the 10 orangutans solved the task and the 4-year-old children also performed poorly. Even so, it is possible that water visibility paired with more advanced cognitive flexibility may have facilitated solving the task for 6- and 8-year-old children.

Another important difference is that children could pour the water from a pitcher in one motion into the tube whereas apes were required to spit several times to be able to get the peanut. The fact that 11 apes in the current study spat water in the tube but only 5 spat enough water to retrieve the peanut suggests that multiple spits (especially if subjects were not very skillful at aiming the water into the tube) may have made the task particularly demanding for apes. We assume therefore that this was the main reason why there is no discrepancy between using the water (pouring) and getting the peanut in the children. All children who used the pitcher also managed to retrieve the peanut at the end. In contrast, apes that had the idea of using water also needed a high amount of persistence in order to get the peanut.

Thus, it is reasonable to assume that the experimental setup might have been more disadvantageous to the apes than the children. Although a more equivalent design would have been desirable, the testing settings and species' natural dispositions made this impractical. In particular, using mouthfuls of water and water pitchers were unfeasible for children and apes, respectively. And not showing children that water was available nearby (and that they were allowed to use it) but providing apes with free access to the dispenser also seemed problematic. Consequently, the performance of the apes compared to that of the children should be taken as a lower-bound estimate of the former's capacities. Future studies could implement procedural modifications that would make the ape and human versions of the task more similar, albeit not identical. For example, children could be required to pour multiple cups of water from an opaque receptacle with water to solve the task, although the experimenter would still have to call attention to the existence of water nearby prior to the test.

In conclusion, we found a remarkable problem-solving ability in chimpanzees and human children. All successful subjects found the solution by themselves, and even though the cognitive affordances that are crucial for this task are not fully understood, the demonstrated behavior can be described as insightful. In addition, we provide suggestive empirical evidence for functional fixedness in chimpanzees—a phenomenon that until now had only been systematically investigated in humans.
